# A Dynamic Proton Bond: MH^+^·H_2_O ⇌ M·H_3_O^+^ Interconversion in Loosely Coordinated Environments

**DOI:** 10.1021/acs.jpclett.2c03832

**Published:** 2023-02-01

**Authors:** Bruno Martínez-Haya, Juan Ramón Avilés-Moreno, Francisco Gámez, Jonathan Martens, Jos Oomens, Giel Berden

**Affiliations:** † Department of Physical, Chemical and Natural Systems, Universidad Pablo de Olavide, 41013 Seville, Spain; ‡ Department of Applied Physical Chemistry, Universidad Autonoma de Madrid, 28049 Madrid, Spain; § Departamento de Química Física, Universidad Complutense, 28040 Madrid, Spain; ∥ Institute for Molecules and Materials, FELIX Laboratory, Radboud University, Toernooiveld 7, 6525ED Nijmegen, The Netherlands

## Abstract

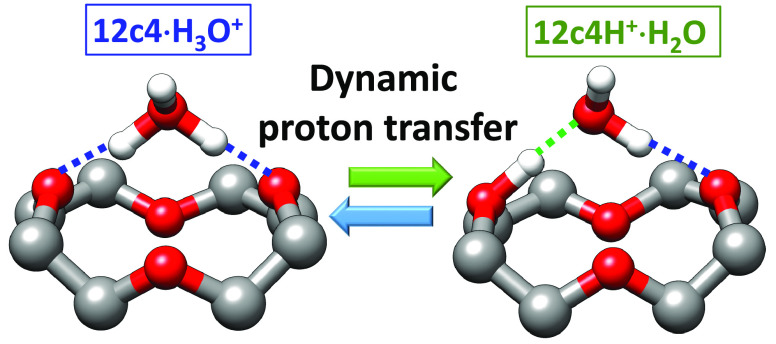

The interaction of organic molecules with oxonium cations within their solvation shell may lead to the emergence of dynamic supramolecular structures with recurrently changing host–guest chemical identity. We illustrate this phenomenon in benchmark proton-bonded complexes of water with polyether macrocyles. Despite the smaller proton affinity of water versus the ether group, water in fact retains the proton in the form of H_3_O^+^, with increasing stability as the coordination number increases. Hindrance in many-fold coordination induces dynamic reversible (ether)·H_3_O^+^ ⇌ (etherH^+^)·H_2_O interconversion. We perform infrared action ion spectroscopy over a broad spectral range to expose the vibrational signatures of the loose proton bonding in these systems. Remarkably, characteristic bands for the two limiting proton bonding configurations are observed in the experimental vibrational spectra, superimposed onto diffuse bands associated with proton delocalization. These features cannot be described by static equilibrium structures but are accurately modeled within the framework of *ab initio* molecular dynamics.

The rationalization of supramolecular behavior in protic environments is a keystone of broad fields in chemical, biological, and materials sciences.^1^ Proton bonding and proton transfer are intrinsically linked to the devious interplay of protonated compounds with their solvation shell.^2^ The mere description of the charge delocalization intrinsic to proton bonding poses considerable challenges to quantum chemistry.^3−6^ The investigation of microsolvated M·H^+^·(H_2_O)_*n*_ complexes of an organic molecule (M) with oxonium cluster ions provides a notable first-principles approach to fundamental aspects of proton interactions and delocalization effects,^7−10^ which then guide the rationalization and modeling of proton bonding in bulk solution.^11−14^ The characterization of even singly hydrated complexes has proven to be of fundamental interest to gain insights into water-mediated proton transfer processes.^7^ This study explores the behavior of hydrated protonated crown ethers. Despite the considerable knowledge accumulated over decades on the supramolecular chemistry of polyether macrocyles,^15^ basic aspects of their ionophoric activity in protic solvents remains under scrutiny. The stabilization of the H_3_O^+^ cation by crown ethers appears to be contradictory with the hierarchy expected from the basicity scale.^16^ For instance, the proton affinity of the ether group (*e*.*g*., 790 and 830 kJ·mol^–1^ for dimethyl ether and diethyl ether, respectively) is substantially higher than that of water (690 kJ·mol^–1^).^17,18^ The native crown ethers considered in this study, 12-crown-4 (12c4), 15-crown-5 (15c5), and 18-crown-6 (18c6), represented in Figure 1, have multiple ether sites and feature even higher net proton affinities, lying within 925–970 kJ·mol^–1^.^17,19^ It is then remarkable to find that water retains the proton, in the form of H_3_O^+^, in its coordinated complexes with the crown ethers. The capture of the proton by a water molecule in the crown ether cavity proceeds upon redistribution of the charge along the coordination bonds, which qualitatively mimics the similar process induced upon hydration of the protonated ether group by a sufficiently large water cluster. It will be shown that the proton migrates from the ether to water as the coordination number increases. In the protonated complex of water and diethyl ether (single coordination), the proton is strongly bound to the ether. In contrast, in the 18c6 complex, the H_3_O^+^ cation is stabilized by a robust symmetric tripodal coordination arrangement. The analogous complexes with the smaller macrocycles 15c5 (nonsymmetric 3-fold coordination) and 12c4 (2-fold coordination) behave as intermediate cases in which the transfer of the proton to the ether groups becomes progressively more facile. In these latter complexes, reversible (ether)·H_3_O^+^ ⇌ (ether)H^+^·H_2_O interconversion takes place recurrently. This work combines infrared multiple-photon dissociation spectroscopy (IRMPD) of the protonated complexes with Born–Oppenheimer molecular dynamics (BOMD) computations to expose the vibrational signatures of proton sharing in these sytems. Details about the methodology employed are provided at the end of the Letter.

**Figure 1:**
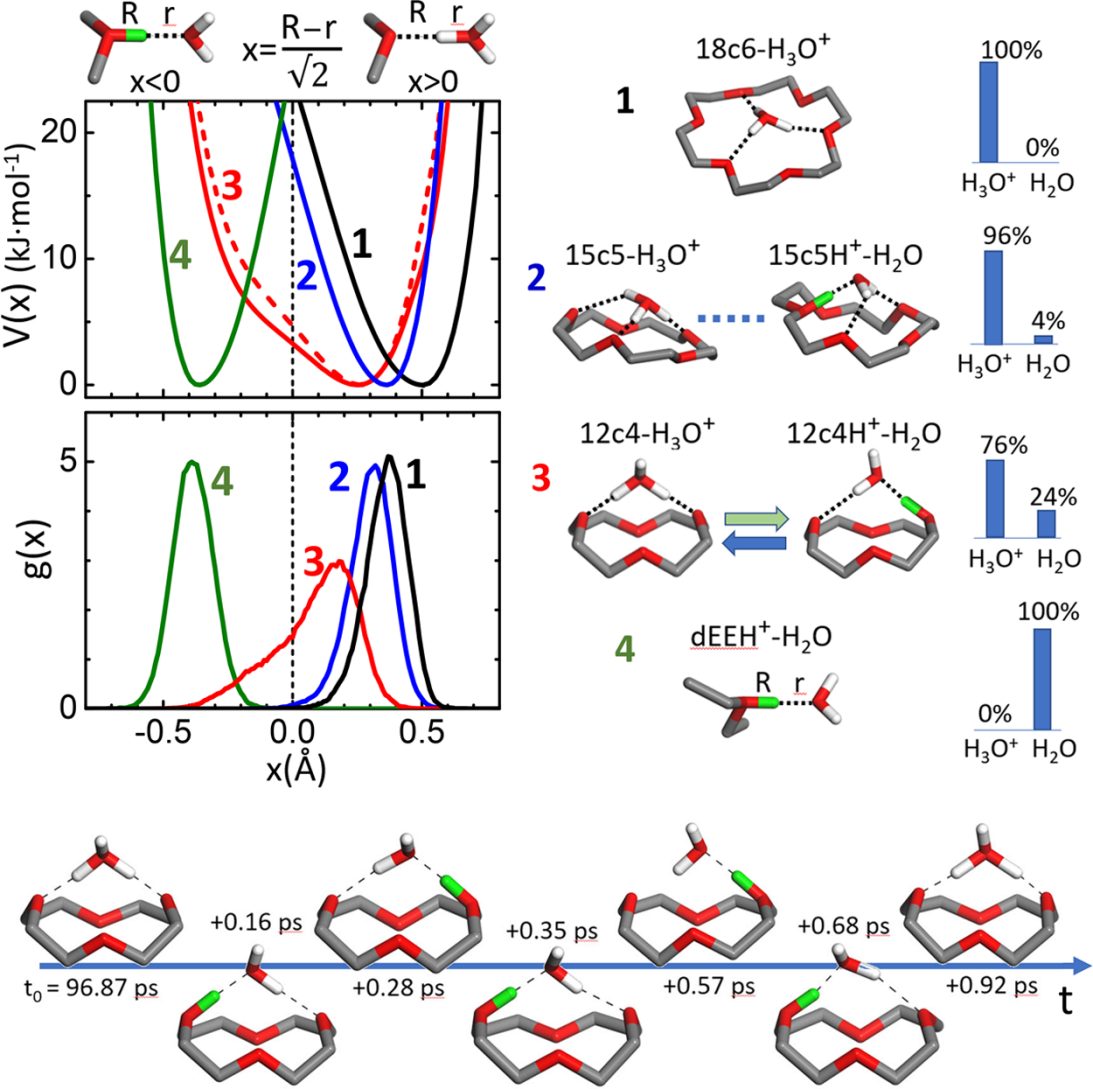
Summary of the conformations adopted by the proton bonded complexes of water with the 18c6 (**1**), 15c5 (**2**), and 12c4 (**3**) crown ethers, and with diethyl ether (dEE, **4**). Left top: B3LYP-D3 relaxed potential energy surfaces, *V*(*x*), for the proton in the O···H^δ+^···O coordinating bonds; for the 12c4 complex, the MP2 computation is also shown (dashed trace). Left middle: BOMD distribution functions of the proton along the coordinating bond, *g*(*x*). Right top: typical conformations and fractions of the BOMD trajectory of each complex in which the asymmetric stretch coordinate takes values *x* > 0 and *x* < 0, leading to effective H_3_O^+^ or H_2_O guests, respectively. Bottom: Illustration of a common event of proton delocalization in the 12c4 complex. For a better visualization, ether-bound protons are represented in green and the H atoms of the methylene macrocyle groups are omitted.

The framework within which proton delocalization occurs in the hydrated crown ether complexes is illustrated in Figure 1, which depicts the effective relaxed potential energy surfaces (PESs) for the proton trapped in between two oxygen atoms, in terms of the asymmetric stretch coordinate, *x*. Also shown are the relative weight in the BOMD trajectories of prototypical configurations of the type (ether)·H_3_O^+^ (hydronium complex, *x* > 0) versus (etherH^+^)·H_2_O (water H-bonded to protonated ether, *x* < 0). The analogous BOMD analysis for the diethyl ether complex (dEEH^+^–H_2_O) is also included for reference. The computed PESs support the above-mentioned trend of migration of the proton from ether to water as the multipodal character of the coordination arrangement increases. Coordination in the diethyl ether complex is accurately described as a protonated ether H-bonded to a neutral water molecule; in this case, the well of the PES is located at high negative values of *x* and the proton remains bound to the ether group, as shown in Figure 1. In the opposite benchmark case, the 18c6 cavity provides a roughly commensurate template for tripodal coordination, leading to a robust stabilization of the H_3_O^+^ cation. Consequently, the well of the PES and the distribution of the proton along the coordinating bonds are centered at high positive values of *x*. Tripodal coordination is conformationally constrained in the asymmetric 15c5 cavity, inducing a shift of the proton distribution toward the ether, with a sizable leak into negative values of *x* (proton located closer to the ether moiety than to water) during ∼4% of the BOMD trajectory. The smaller 12c4 cavity hinders the optimization of three coordination bonds and imposes a bipodal coordination to hydronium. This has profound effects on the dynamics of proton bonding. The well of the PES for the proton in the 12c4 complex is appreciably broadened with respect to the larger crown ethers and extends to negative values of *x*. Consistently, the proton is significantly delocalized between the ether and water moieties. The well is asymmetric, and the 12c4·H_3_O^+^ arrangement is still favored with respect to 12c4H^+^·H_2_O, by an average ratio of 0.74/0.26 according to the BOMD computation.

The efficient exchange of the proton between water and ether moieties in the 12c4 complex revealed in this study is intriguing. The dynamic picture that emerges is that of a recurrent M·H_3_O^+^ ⇌ MH^+^·H_2_O interconversion promoted by the two-fold coordination in the 12c4 macrocyle. Figure 1 illustrates a typical progression of proton transfer between water and the crown ether during a 1 ps time window. It becomes apparent that the limiting protonated ether 12c4H^+^–H_2_O and protonated water 12c4–H_3_O^+^ configurations are dynamically unstable and the proton is prone to diffuse between water and ether. Interestingly, proton sharing alternates between the two water–ether coordinating bonds, leading to a Grotthuss-like rearrangement of the water covalent bonds. An extended representation of the proton-sharing dynamics is provided in Figure S1 of the Supporting Information. Note that the time scale of ∼0.1 ps for the observed proton exchanges is roughly 1 order of magnitude slower than the period of vibration of the O–H stretching modes of water or hydronium, so that the two types of motion are uncoupled.

It is timely to stress that crown ether flexibility increases with size, which commonly leads to active ring puckering dynamics (*i*.*e*., changes in the dihedral angles along the cyclic backbone).^21^ In fact, rich configurational dynamics were observed for the protonated forms of the 12c4, 15c5, and 18c6 macrocyles.^6^ Interestingly, the 18c6 backbone is fixed to the roughly planar *C*_3*v*_ configuration by its commensurate coordination with H_3_O^+^. The loss of symmetry makes the 15c5–H_3_O^+^ complex more labile, allowing for puckering in the crown ether ring and for changes in the coordination sites.^20^ While the 12c4 complex is less prone to puckering due to the shorter ring length, changes in dihedral angles transiently leading to higher-energy conformations are as well observed in the computed dynamics. These puckering effects are illustrated in Figure S2 of the Supporting Information, which shows two typical configurations of the 15c5 and 12c4 complexes connected by ring puckering, along with the associated probability distribution of one of the COCC dihedral angles involved in the transformation as derived from the BOMD trajectory.

The IRMPD vibrational spectra measured for the three ion complexes are compiled in Figures 2 and 3, along with computational counterparts. The broad spectral range covered by the experiments samples a variety of stretching and bending modes of the host and guest moieties, which allows the exposure of signatures of the dynamic proton-bonding. A qualitative assignment of the main vibrational bands observed is provided in the Supporting Information. The modes of the complex that are dominated by vibrational motions of the macrocycle backbone groups (C–C, C–O, C–H stretching, CH_2_ twisting, wagging, and scissoring) produce comparably narrow-band features that are labeled by lower-case letters a–g in Figure 2. The bending (twisting, umbrella, and scissoring) and stretching modes of H_3_O^+^ lead to broader, diffuse band structures (labeled T, U, B, S, and W in Figures 2 and 3) that partially overlap with the macrocycle bands. Two outstanding features of the recorded spectra can be pointed out. First, the stretching modes of the O–H^δ+^ bonds sustaining the proton bonding in the complexes give rise to a particularly broad spectral feature extending over the 1500–3500 cm^–1^ range (band S). Second, only for the 12c4 complex, vibrational signatures of both H_3_O^+^ and neutral H_2_O are remarkably detected at >3500 cm^–1^ (band W). A detailed analysis of this latter feature in terms of the O–H^δ+^/O–H stretching mode contributions to the IRMPD spectra is provided below (Wa, Ws, and Wf bands in Figure 3).

**Figure 2:**
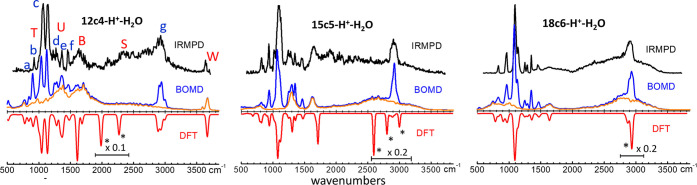
Experimental IRMPD spectra and computational (BOMD, DFT) IR spectra for isolated proton-bonded complexes of water with crown ethers 18c6, 15c5, and 12c4. The broad spectral range investigated exposes narrow bands from stretching and bending modes of the macrocycle (CH_2_CH_2_O) groups (labeled a–g) and partially overlapping diffuse bands from modes of the H_2_O·H^+^ moiety (labeled T, U, B, S, and W). See Table S1 of the Supporting Information for qualitative mode assignments. The BOMD spectra (blue trace, full spectrum; orange trace, bands associated with vibrational motions of the H_2_O·H^+^ moiety only) show diffuse O–H^+^ stretching features (bands S) in good agreement with experiment, while the analogous DFT bands are strong and localized (peaks marked with asterisks, scaled in intensity by the indicated factors).

**Figure 3:**
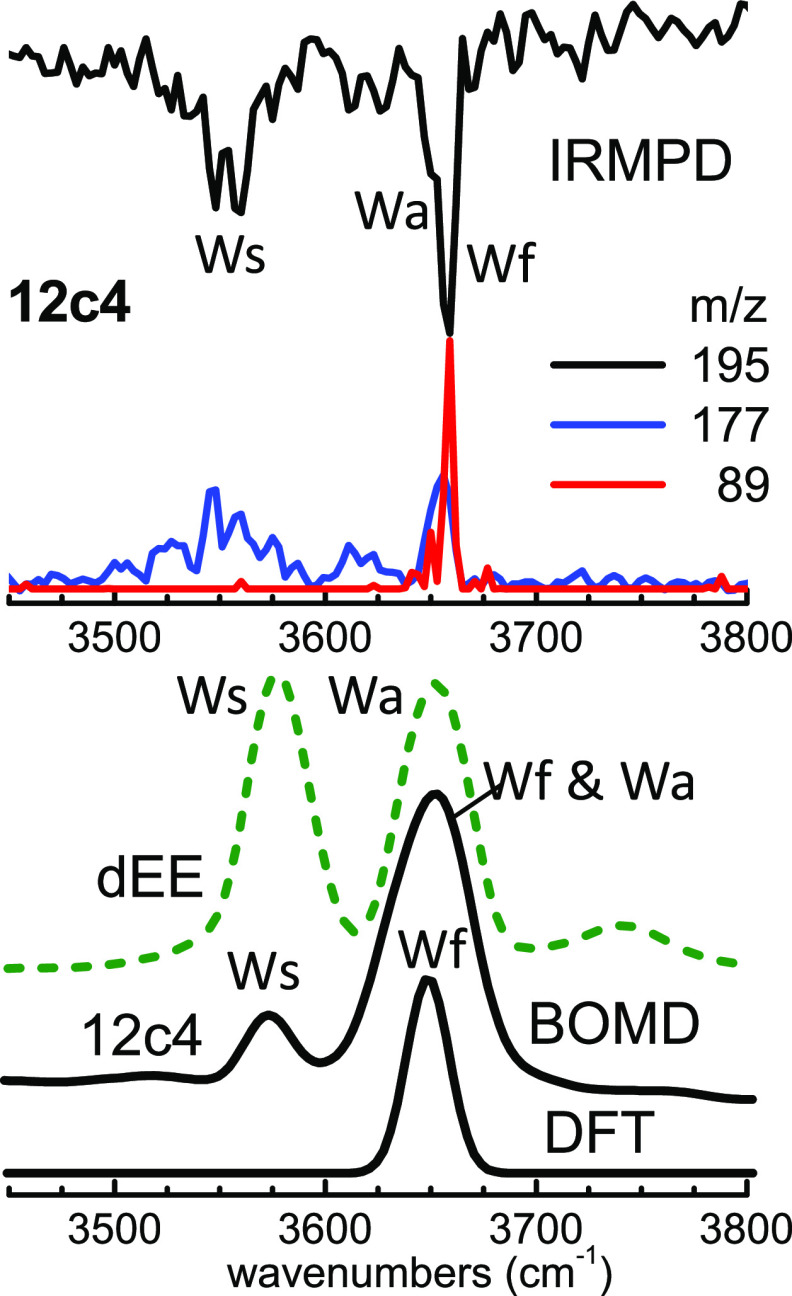
Detailed analysis of H_3_O^+^ and H_2_O signatures in the IRMPD spectrum of the 12c4 complex. Top: IRMPD signals obtained with the OPO laser at the masses of the full complex (*m*/*z* = 195, depletion spectrum) and of two photofragments, namely, the protonated macrocycle 12c4H^+^ (*m*/*z* = 177, blue trace) and the protonated half macrocycle (CH_2_CH_2_O)_2_H^+^ (*m*/*z* = 89, red trace). Bottom: BOMD and DFT spectra for the 12c4 complex (full black traces), and BOMD spectrum for the diethyl ether complex (dEE, dashed green trace). See text and the Supporting Information for an assignment of the Ws, Wa, and Wf bands.

The assignment of the vibrational bands observed experimentally is nicely supported by the good overall agreement with the BOMD-computed IR spectra for the three complexes. The interpretation of the diffuse contributions of the hydronium and water modes to the overall IR spectrum of each complex is aided by the deconvolution of the motion of the H_2_O·H^+^ moiety in the BOMD trajectory, leading to the traces depicted in orange in Figure 2. The particularly broad S bands observed for the three complexes are associated with the stretching vibrations of the coordinating bonds, which lays out a scenario of a marked entanglement of proton sharing with the positional and orientational dynamics of the H_3_O^+^ guest cation.^5,6,20,22−26^ Proton delocalization presumably induces a dynamic rearrangement of electronic density in the complex, leading to a broad ensemble of coordinating bond strengths and effective stretching frequencies. Notably, a qualitatively similar, though narrower, diffuse S band was observed by Johnson and co-workers in the vibrational spectrum of the (D_2_-tagged) 18c6–H_3_O^+^ complex at temperatures as low as 10 K,^26^ indicating that proton delocalization remains active well below room temperature. It can also be noted that the bending vibrational modes of the H_2_O·H^+^ moiety (T, U, and B) induce diffuse band structures in the fingerprint region of the spectrum (500–2000 cm^–1^). These bands are notably broader in the 12c4 complex than in the 15c5 and 18c6 ones. This is consistent with the more marked distortion of the supramolecular structure resulting from the active proton transfer between water and the crown ether for 12c4.

It is timely to stress that the spectrum observed for the protonated complexes of water with the crown ethers cannot be reproduced with static quantum-chemical modeling approaches (*e*.*g*., at the DFT level). Static computations yield individual equilibrium configurations, of the form (ether)·H_3_O^+^ for the most stable configuratons of the present crown ether systems, which do not capture the dynamic nature of the coordination networks. The spectra shown in Figure 2, predict nevertheless fundamental frequencies that match fairly well the relative positions of the host macrocycle backbone modes and that fall within the central region of the broad bands associated with the guest vibrational modes. Note that the strong and localized DFT-computed S band transitions (marked with asterisks) are scaled in intensity in Figure 2 for a better visualization of the remaining vibrational bands.

While the BOMD computation reproduces the experimental IRMPD spectra with remarkable accuracy, some differences are also apparent, in particular for the envelope of the proton-stretching S band. The best agreement is found for the 18c6 complex, which provides the most rigid coordination arrangement with H_3_O^+^. In this case, the position and width of the S band are roughly coincident in experiment and computation. For the 15c5 complex, the S band appears to be broader and more spread toward the lower-energy spectral region in the IRMPD spectrum compared to the BOMD prediction. As argued above, this is the most labile complex among the three ones here investigated. The agreement is somewhat recovered for the 12c4 complex, yet sizable differences persist for the relative intensity of the S band with respect to other spectral bands. The O–H^+^ stretching vibrational mode associated with the S band can be expected to be particularly sensitive to configurational fluctuations altering the proton bonding network. Such a complex scenario is not completely captured within the B3LYP/DZVP framework of the present BOMD computations. At a static level, the B3LYP functional appears to reproduce correctly the electronic structure and interactions of proton bonding in regions close to minimum energy configurations, as suggested by the good comparison with the MP2 computation for the potential energy surface shown in Figure 1. However, limitations seem to emerge at a dynamic level, where the description of proton bonds within dynamically changing host–guest geometries probably demands more accurate, yet cost-effective, functionals and/or basis sets.

Band W observed experimentally only for the 12c4 complex deserves specific consideration. Figure 3 depicts the IRMPD signals obtained for this band with the high-resolution OPO laser in the 3400–3800 cm^–1^ range. Shown are the depletion channel (loss of parent ion signal at *m*/*z* 195) and the fragmentation channels leading to the protonated macrocycle 12c4H^+^ (*m*/*z* 177), or half macrocycle (CH_2_CH_2_O)_2_H^+^ (*m*/*z* 89). Two IRMPD band structures are observed in this region, at 3550 and 3660 cm^–1^. The BOMD computation correctly reproduces the presence of the two bands and suggests that they are associated with the contributions of distinct modes of H_3_O^+^ and H_2_O moieties. Neutral water is monitored in this spectral region through the symmetric and asymmetric stretching modes, which we here denote Ws and Wa, respectively. Figure 3 shows that the BOMD computation for the dEEH^+^–H_2_O complex displays neat Ws and Wa water bands, due to the robust protonation at the ether group. The low-energy band in the IRMPD spectrum of the 12c4 complex, at 3550 cm^–1^, is consistently assigned by the BOMD computation to the symmetric O–H stretching mode of water. The presence of this band is remarkable, as it can only be traced back to proton transfer from hydronium to the ether ring, leading to the transient formation of neutral water. The asymmetric O–H stretching mode (Wa) then contributes to the higher-energy band observed in the spectrum at 3660 cm^–1^. This latter band is sharper and more intense in the IRMPD experiment than the former one, as it receives an additional contribution (denoted Wf) from the stretching mode of the free O–H^δ+^ bond of hydronium. The Wf band is hence reminiscent of the dominant two-fold 12c4–H_3_O^+^ coordination. It becomes apparent that the IRMPD spectrum of the 12c4 complex shows spectral features of both water and hydronium thereby suggesting (ether)·H_3_O^+^ ⇌ (ether)H^+^·H_2_O dynamic interconversion. Figure 3 shows that the static DFT computation only predicts the presence of the Wf band, whereas the BOMD computation reproduces with remarkable accuracy the spectral signatures of the dynamic system, including the bands associated with the H_2_O moiety.

Interestingly, we find experimentally that IRMPD on the stretching bands of water in the 12c4 complex leads exclusively to a prompt water loss yielding 12c4H^+^. This suggests that the excitation of the stretching modes of H_2_O in configurations of the type 12c4H^+^·H_2_O is not followed by a sufficiently rapid vibrational energy redistribution within the complex, so that water detachment occurs before any appreciable heating of the crown ether takes place. In contrast, excitation of the Wf hydronium stretching mode induces crown ether backbone fragmentation through C–O cleavage to yield (CH_2_CH_2_O)_2_H^+^, in addition to water detachment. This is in fact the IRMPD process observed in all vibrational bands measured in the experiments, with the notable exception of the stretching bands of neutral water. The observation of mode-dependent fragmentation channels is indicative of nonergodic behavior, which is unusual in ionic systems of the size of the 12c4 complex.^28^

The behavior observed in this study for the native crown ethers plausibly finds analogies in related molecular substrates with polar O atom groups. The coexistence of M·H_3_O^+^ and MH^+^·H_2_O configurations can be expected to emerge in systems with sufficiently weak coordination, embedded in water-poor environments in which an extensive hydration cluster cannot be formed. In such cases, the structure of the complex is not static and can only be rationalized within dynamic modeling schemes. In particular, the broadening of spectral bands observed in the IRMPD spectra are intrinsic to the nature of loose proton bonding frameworks and rationalizes early failures to explain the spectral features of these systems with static DFT or MP2 computations.

## Materials and Methods

### IRMPD Experiments

Infrared multiple photon dissociation (IRMPD) vibrational spectra were recorded for the proton-bonded complexes of water with the crown ethers 12c4, 15c5, and 18c6 (*m*/*z* = 195, 239, and 283, respectively). The complexes were produced by electrospray ionization of aqueous solutions of the crown ethers at concentrations of ∼100 μM, with added trifluoroacetic acid. The resulting cationic complexes were isolated in a quadrupole ion trap mass spectrometer (Bruker AmaZon Speed) at room temperature for spectroscopic interrogation.^27,29^

The IRMPD experiments covered an uncommonly broad spectral range, 600–3800 cm^–1^, in order to probe vibrational modes of the host macrocycle as well as of the H_3_O^+^/H_2_O guest. The fundamental and third harmonic output of the free electron laser were employed to cover the 600–2000 cm^–1^ (20–130 mJ/pulse) and 1800–3700 cm^–1^ (5–20 mJ/pulse) spectral ranges, respectively (spectral bandwidth ∼0.5% of the central IR frequency). The ions were irradiated with a single FELIX pulse, which consists of a 5 μs long train of micropulses at a repetition rate of 1 GHz. The pulse energy was attenuated to prevent excessive precursor ion depletion and formation of fragment ions below the low mass cutoff of the quadrupole ion trap.^30^ The OH stretching region was explored in greater detail in additional measurements at higher spectral resolution around 3400–3800 cm^–1^ using a high repetition rate optical parametric oscillator (OPO) (LaserSpec, Belgium, spectral bandwidth 0.5 cm^–1^, 5 nJ/pulse, 80 MHz repetition rate, 100 ms irradiation).^31^

When the laser frequency matches a vibrational transition of the isolated ion complex, multiple photon absorption occurs, resulting in resonant fragmentation. The main product fragment detected in the IRMPD process was the protonated crown ether, resulting from water loss in the parent complex. Weaker signals from crown ether fragments of the form (CH_2_CH_2_O)_*n*_H^+^ were observed as well. Only for one particular band of the 12c4 complex (band Wf) was the (CH_2_CH_2_O)_2_H^+^ fragment more intense than the protonated crown ether. The IRMPD spectrum was produced from the precursor intensity (*I*_p_) as a depletion experiment, and from the product ion intensities (*I*_f_), in this case by plotting −ln(*I*_p_/[*I*_p_ + *I*_f_]) as a function of the IR frequency. Linear normalization was applied to account for changes in the laser energy during scans.^30^

### Computations

Density functional theory (DFT) at the B3LYP-D3/6-311++G(d,p) level (D3 stands for Grimme’s D3BJ dispersion corrections) was employed to assess the structural features of the configurations of lowest energy of the crown ether complexes. The computations were also performed for the complex of protonated diethyl ether with water as reference. Candidate structures were generated by means of simulated annealing with different empirical force fields. All the equilibrium structures produced at the DFT level were of the (crown)-H_3_O^+^ form, hence, with a stable hydronium guest cation. Complementary *ab initio* MP2 computations with the same basis set did not alter significantly the energetic and spectral features predicted by DFT. Moreover, the use of larger basis sets, up to 6-311++G(2df,2pd), similarly led to equivalent DFT results within the scope of our study. Harmonic IR spectra were generated by convoluting the normal modes of vibration of the DFT conformers with a Gaussian line broadening of 30 cm^–1^ fwhm. For comparison with experiment, the DFT spectra were scaled by factors of 0.98 and 0.95 at wavenumbers below and above 2000 cm^–1^, respectively.^32^

Born–Oppenheimer molecular dynamics (BOMD) computations of the same proton-bound complexes were performed within the framework of the CP2K code.^33^ A number of reviews provide comprehensive descriptions of the BOMD and related Carr–Parinello molecular dynamics methods.^34−36^ The present BOMD computations employed the B3LYP functional with the double-ζ DZVP basis set; the D3BJ dispersion correction; and the Goedecker, Teter, and Hutter pseudopotentials.^37^ The cutoff radius for the pair potential was set to 12.5 Å, and a cubic cell of side length 25 Å was employed for the isolated complex. The complexes were equilibrated in the *NVT* ensemble at 350 K, with the CSVR thermostat (canonical sampling through velocity rescaling) for 5 ps. Subsequently, a computation in the *NVE* ensemble was performed to monitor the dynamics of the complexes over 150 ps. With the computing resources available for this investigation (parallel computation on a 40-core 2.4 GHz Intel Xeon processor), the molecular dynamics could be calculated over 15–20 ps per week. During the *NVE* stage, the temperature fluctuated around the average value of ∼350 K with a standard deviation of 30 K. Infrared spectra were produced with the TRAVIS analyzer package^38^ from the Wannier center coordinates produced during the BOMD trajectories. The BOMD spectra were scaled for comparison with experiment by factors 1.0 (no scaling) and 0.96 at wavenumbers below and above 2000 cm^–1^, respectively. Unlike the static DFT computations, the BOMD trajectories unveiled mixed (crown)-H_3_O^+^ and (crown)H^+^-H_2_O configurations as described below.

Seeking to gain insights on the interactions driving proton bonding, relaxed potential energy surfaces along the asymmetric stretch of the intramolecular proton bond were computed at the B3LYP-D3 and MP2 levels (both methods led to similar results). In these calculations, one O···H^+^ distance in a proton bond is scanned while all other degrees of freedom of the molecular system are allowed to equilibrate to their configuration of minimum energy.

## Supporting Information Available

Qualitative assignment of vibrational bands observed in the IRMPD spectra; illustration of events of proton exchange in the 12c4 complex and of ring puckering in the 15c5 and 12c4 complexes observed in the BOMD computations (PDF)
Transparent Peer Review report available (PDF)
