# Supplementary material for: A Dynamic Proton Bond: MH+·H2O ⇌ M·H3O+ Interconversion in Loosely Coordinated Environments
Source: J Phys Chem Lett. 2023 Feb 1;14(5):1294–300. doi: 10.1021/acs.jpclett.2c03832 (PMC9923742; doi:10.1021/acs.jpclett.2c03832)

Supporting Information to: A Dynamic Proton Bond: $MH^+ \cdot H_2O \rightleftharpoons M \cdot H_3O^+$ Interconversion in Loosely Coordinated Environments

Bruno Martínez–Haya[a],* Juan Ramón Avilés–Moreno[b], Francisco Gámez[c], Jonathan Martens[d], Jos Oomens[d], and Giel Berden[d]

[a] *Department of Physical, Chemical and Natural Systems, Universidad Pablo de Olavide, 41013 Seville, Spain.*

[b] *Department of Applied Physical Chemistry, Universidad Autonoma de Madrid, 28049, Madrid, Spain.*

[c] *Departamento de Química Física, Universidad Complutense, 28040 Madrid, Spain.*

[d] *Radboud University, Institute for Molecules and Materials, FELIX Laboratory, Toernooiveld 7, 6525ED Nijmegen, The Netherlands.*

E-mail: bmarhay@upo.es

Vibrational Band Assignments

Table S1: Qualitative assignment of the fundamental modes contributing to the vibrational bands observed in the IRMPD spectra of the ptrotonated complexes of water with the crown ethers 12c4, 15c5 and 18c6. The spectra are represented in Figs 2 and 3 of the paper. The broad spectral range investigated exposes narrow bands from stretching and bending modes of the macrocycle (CH_2CH_2O) groups (labelled a-g) and partially overlapping diffuse bands from modes of the $H_2O{\cdot}H^+$ moiety (labelled T, U, B, S, W). See Fig. 3 for a detailed analysis of the decomposition of the W band into H_3O^+ (Wf) and H_2O (Wa, Ws) contributions.

Band	mode assignment
**a**	CH_2 rocking; COC bending
**b**	C–C stretching;<br>CH_2 rocking, COC bending
**c**	C–O stretching; C–C stretching,<br>CH_2 rocking, OCCO torsions,
**d**, **e**, **f**	CH_2 twisting, wagging, bending
**g**	C–H stretching
**T**	H_3O^+ twisting
**B**	H_3O^+/H_2O scissoring
**U**	H_3O^+ umbrella
**S**	str. of coordinated O-$H^{\delta+}$
	**Wa**: asymmetric O-H str. of H_2O
**W**	**Ws**: symmetric O-H str. of H_2O
	**Wf**: str. of 'free' O-$H^{\delta+}$ of H_3O^+

Insights into proton exchange in the 12c4·H^+·H_2O complex

Figure S1: Illustration of reversible proton transfer events in the BOMD computation of the 12c4 complex. The top panel represents the time evolution of the asymmetric stretch coordinate x, associated with each of the two O$\cdots$H$^{\delta+}\cdots$O coordinating bonds (red and blue traces). Note that x<0 and x>0 are associated to protons more strongly bound to the ether and to the water moieties, respectively (see Fig. 1 of the paper for the definition of x). A magnified time window and a set of snapshots are shown for a detailed description of recurrent events of proton transfer occurring within a time span of 1 ps. The proton is shown in green colour when it is bound to the crown ether; the H atoms of the methylene macrocyle groups are omitted for a better visualization.

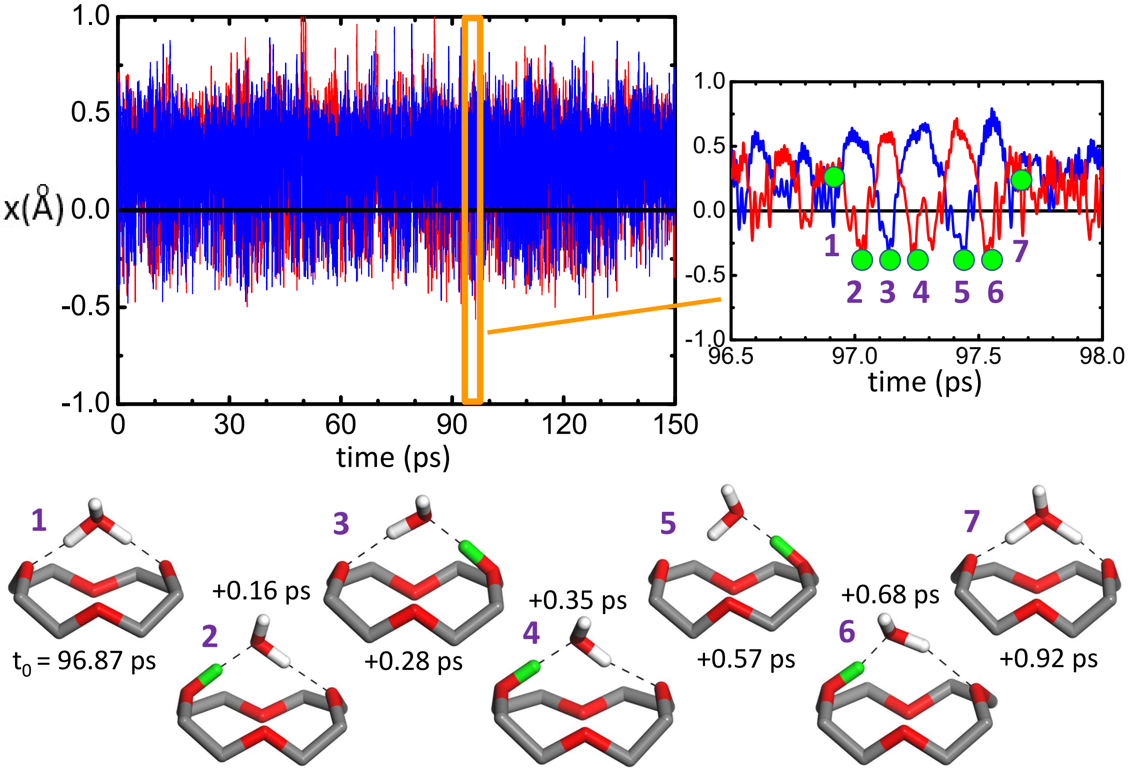

Ring puckering effects in the 15c5 and 12c4 complexes

Figure S2: Illustration of ring puckering effects observed in the BOMD computations of the 15c5 and 12c4 complexes. For each complex, two typical conformations (labelled I and II) are shown which are connected by changes in backbone dihedral angles. A COCC dihedral angle Φ, corresponding to the ring segment highlighted in yellow, is chosen to monitor the structural change. The distribution P(Φ) resulting from the BOMD trajectory for each complex is depicted in the bottom panels. Each peak in the distribution corresponds to one of the two types of configurations. Note also the change in proton bonding coordination partners in the two configurations of the 15c5 complex

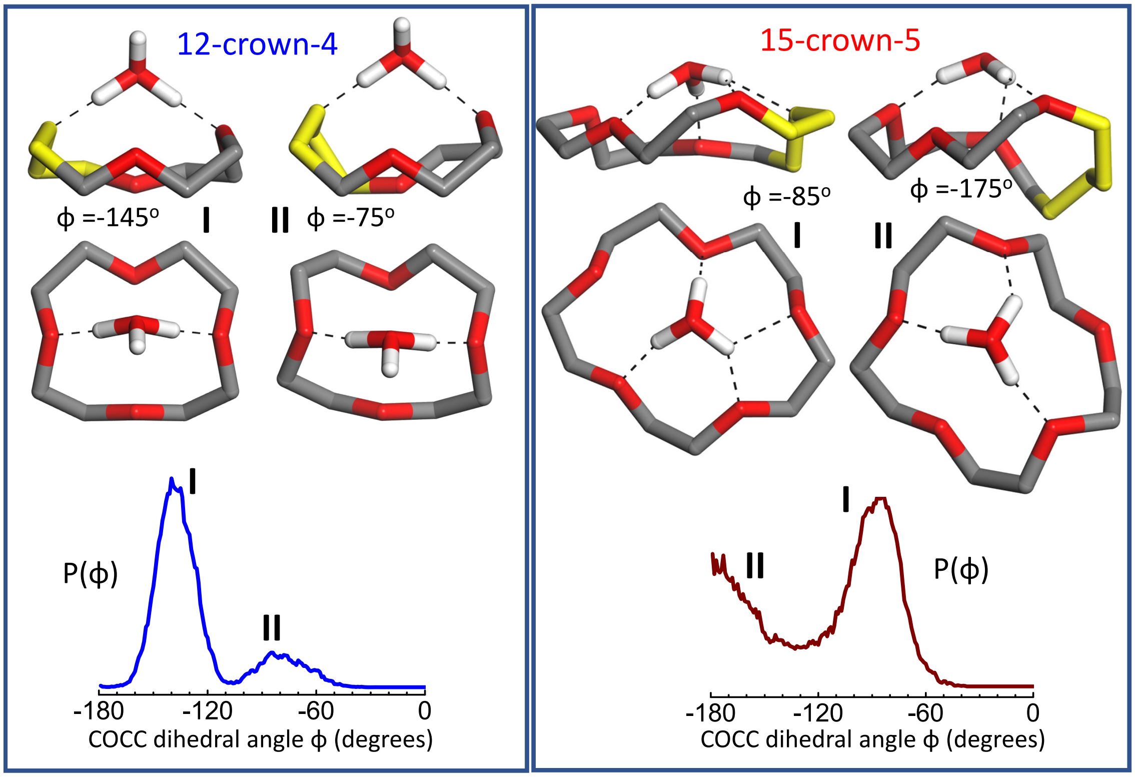

Supplement: Supplementary file 1 — jz2c03832_si_001.pdf [file jz2c03832_si_001.pdf]
