# Supplementary material for: A Dynamic Proton Bond: MH+·H2O ⇌ M·H3O+ Interconversion in Loosely Coordinated Environments
Source: J Phys Chem Lett. 2023 Feb 1;14(5):1294–300. doi: 10.1021/acs.jpclett.2c03832 (PMC9923742; doi:10.1021/acs.jpclett.2c03832)
Supplement: Supplementary file 2 — jz2c03832_si_002.pdf [file jz2c03832_si_002.pdf]

jz-2022-03832a.R1

Name: Peer Review Information for "A Dynamic Proton Bond: $MH^+ \cdot H_2O \rightleftharpoons M \cdot H_3O^+$ Interconversion in Loosely Coordinated Environments"

First Round of Reviewer Comments

Reviewer: 1

Comments to the Author

This manuscript reports the observation of proton delocalization in isolated room-temperature protonated crown ether - water complexes by IR multiple-photon dissociation (IRMPD) spectroscopy, interpreted by Born-Oppenheimer Molecular Dynamics (BOMD) simulations and static DFT calculations.

This study is interesting and well presented. It is publishable subject to the following revisions:

1) Although experiment and theory compare well enough to support conclusions, I regret that the authors claim an "excellent agreement", whereas obvious differences exist and deserve a short comment. This is the case of band S for complexes with 12c4 and 15c5 (Fig. 2) whose location and shape do not look well predicted by BOMD simulations. Does the method used (B3LYP-D3BJ/DZVP) enable the authors to predict accurately enough the PES of these systems, including the X value of its minimum and the broadening towards negative values (Fig. 1), which must be critical parameters for this band prediction? I believe the authors miss an opportunity to point out a limitation of their theoretical method, without taking away the nice achievement of BOMD simulations.

2) The authors did not say anything about ring puckering. They should explain why they did not consider it.

3) Information about the experimental and theoretical temperatures are in the SI but should be placed in the main text.

4) Figure 1 : please precise in the caption B3LYP-D3 (or MP2) for the PES shown.

Author's Response to Peer Review Comments:

The Journal of Physical Chemistry Letters
Editorial office

Nijmegen (The Netherlands), January 20, 2023

Dear Editor,

Enclosed please find **the revised version** of the manuscript entitled "*A dynamic proton bond: $MH^{+}\cdot H_2O$<->$M\cdot H_3O^{+}$ interconversion in loosely coordinated environments*" by Bruno Martínez-Haya, Juan R. Avilés-Moreno, Francisco Gámez, Jonathan Martens, Jos Oomens and Giel Berden, which we submit for publication in The Journal of Physical Chemistry Letters

In this revised manuscript, we believe to have accounted for the remarks included in the review report. In addition, we have followed the editorial guidelines related to formatting and layout, mainly by producing the Latex file under the achemso environment. All changes in the text are highlighted in red color in the manuscript and supporting information.

In the following, we provide point-by-point responses to the issues raised in the review report.

1) *Although experiment and theory compare well enough to support conclusions, I regret that the authors claim an "excellent agreement", whereas obvious differences exist and deserve a short comment. This is the case of band S for complexes with 12c4 and 15c5 (Fig. 2) whose location and shape do not look well predicted by BOMD simulations. Does the method used (B3LYP-D3BJ/DZVP) enable the authors to predict accurately enough the PES of these systems, including the X value of its minimum and the broadening towards negative values (Fig. 1), which must be critical parameters for this band prediction? I believe the authors miss an opportunity to point out a limitation of their theoretical method, without taking away the nice achievement of BOMD simulations.*

Response: The limitations of the BOMD framework, specifically for the accurate prediction of the broad S band (stretching of the proton bond), are now stressed and discussed in the light of the dynamics observed for the 18c6, 15c5 and 12c4 complexes (pages 10-11). BOMD performance is most reliable for the most rigid 18c6 complex, while it appears to be less accurate for the more flexible 15c5 complex and, to some extent, also for the 12c4 complex. The comparison of B3LYP vs. MP2 proton bonding interactions (Fig. 1) and the discussion of ring puckering (Fig. FS2), incorporated in the light of remarks 2 and 4 below, also contribute to rationalize the accuracy of the present BOMD computations, as discussed in the revised version of the paper.

2) The authors did not say anything about ring puckering. They should explain why they did not consider it.

Response: This is a good point. We have incorporated a concise discussion on ring puckering in the main text (pages 6-7 of the revised manuscript). Complementary to this discussion, a graphical illustration of puckering events has been included as Supporting Information (Figure FS2). Ring puckering is of relevance in the most labile 15c5 complex, partly also in the 12c4 complex, which also contributes to rationalize the accuracy of the BOMD prediction for the S band (remark 1 above). We have included an additional paragraph discussing the implications of this flexibility

3) Information about the experimental and theoretical temperatures are in the SI but should be placed in the main text

Response: The methodological section has been moved to the main text, as requested.

4) Figure 1 : please precise in the caption B3LYP-D3 (or MP2) for the PES shown.

Response: The caption of Fig. 1 has been modified accordingly. Also importantly, we have slightly revised Fig. 1 to include both B3LYP and MP2 potential energy surfaces for the 12c4 complex. The direct comparison of both computational approaches is useful for the assessment of the accuracy of the B3LYP method, as requested in remark 1 of the review report.

We trust that this revised version is suitable for publication in JPC Lett.

With best regards,

Bruno Martínez Haya (corresponding author)
Professor of Physical Chemistry
Universidad Pablo de Olavide (Seville, Spain)
